# From effectors to effectomes: Are functional studies of individual effectors enough to decipher plant pathogen infectious strategies?

**DOI:** 10.1371/journal.ppat.1009059

**Published:** 2020-12-03

**Authors:** Noe Arroyo-Velez, Manuel González-Fuente, Nemo Peeters, Emmanuelle Lauber, Laurent D. Noël

**Affiliations:** LIPM, Université de Toulouse, INRAE, CNRS, Castanet-Tolosan, France; University of Utah, UNITED STATES

Effector proteins of plant pathogens are key virulence determinants which can be secreted in the apoplast or translocated inside plant cells where they subvert host immunity and physiology to the pathogen’s benefit [1]. In some specific plant accessions, effector proteins may also be detected by plant immune receptors and trigger strong specific resistance [2–4].

## Achievements and limits of current effectors studies in plant pathogens

**Fig 1:**
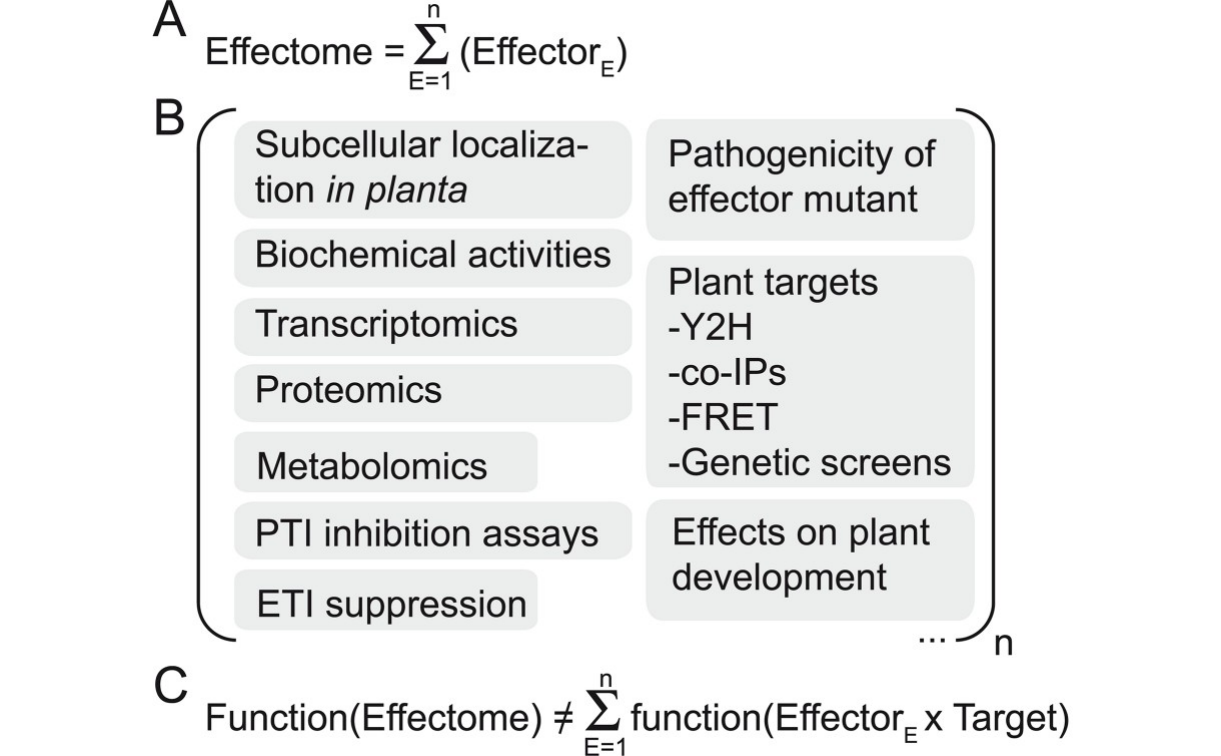
Functions achieved by an effectome are more than the sole addition of the individual effector functions. (A) The effectome is the sum of the n individual effectors from a single pathogen strain. (B) Examples of functional studies which can be conducted for each of the n effector proteins of a given effectome. (C) Due to functional redundancies and epistatic interactions, the effectome function is different from the sum of individual effector functions. Importantly, effector and effectome functions will depend on the composition and diversity of effector targets present in the plant species and accession considered. co-IPs, co-immunoprecipitations; ETI, effector-triggered immunity; FRET, Förster resonance energy transfer; PTI, PAMP-triggered immunity; Y2H, yeast two-hybrid.

**Fig 2:**
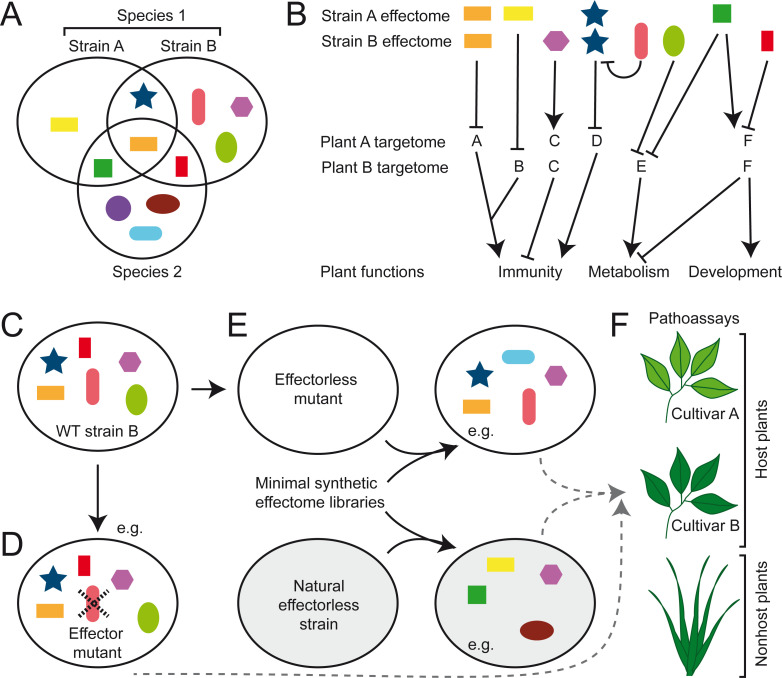
Diversity of both the microbial effectome and the plant target repertoire impacts the function of the effectome. (A) Effectomes are diverse at the intra- and interspecific levels. (B) Individual effectors can have 1 or multiple plant targets with either positive (arrowheads) or negative (blunt arrows) impacts. Hubs are plant proteins or functions which are targeted by multiple effectors. Some effectors might directly or indirectly affect the function of other effectors. To date, plant functions targeted by effectors are immunity, physiology, and metabolism. Distinct plant targets are affected depending on the pathogen effectome. Target diversity implies that different plant accessions will respond differently to distinct effectomes. (C–E) Schematic representation of possible genetic manipulations of effectomes. The effectome of WT strain B (C) can be genetically manipulated by deleting individual (D) or multiple effector genes yielding an effectome mutant (E). Effectorless strains found in the environment can also be used and complemented with the appropriate effector secretion-translocation machinery if missing. Examples of random or informed libraries corresponding to an effector combinatorial originating from strain B or any other strain could be reintroduced in an effectorless strain (E) and tested for functional complementations on host or nonhost plants of multiple cultivars (F). Each symbol represents a distinct effector produced by the pathogen. Members of a given effector protein family are represented with the same shape but different colors. WT, wild-type.

A pathogen’s effectome (sometimes also referred to as effectorome) is the repertoire of all its effector proteins (Fig 1A). To date, most effector proteins are studied individually, omitting the broader context in which they function as the effectome. Size and composition of effectomes vary greatly between pathogens, including at the intraspecific level, ranging from as little as 4 in *Erwinia amylovora* to hundreds of effector proteins per isolate in some fungi, nematodes, and oomycetes (Fig 2A) [5–14]. These differences influence pathogen’s virulence, lifestyle, and host range [15–17]. Known effector functions are the result of a combination of experimental approaches, often low throughput and based on *in vitro* or heterologous systems (Figs 1B, 2C and 2D) [18–21]. Some effectome-scale screens have been conducted, but these are still a compilation of individual effectors studies and thus present the same limitations as smaller-scale studies [22–28].

## Evidences for effector–effector interferences within effectomes

Studies of individual effector proteins intrinsically overlook their coordinated functions due to functional redundancy [29–31], expression patterns dependent on infection stages or plant organ [32–34], and epistatic interactions within effectomes [35–40] (Fig 2B). Therefore, effectome functions are usually not the sum of the individual effector functions (Fig 1C), and dedicated experimental approaches would be needed to determine how effectomes function as a whole. Prerequisites are the knowledge of the effectome composition, an experimentally manageable effectome size, and a genetically amenable pathogen. Consequently, functional characterization of effectomes is most advanced in bacteria [30,35,41,42] and developing at an ever increasing pace in fungi or nematodes thanks to powerful genome-editing tools [43,44] and the frequent clustering of effector genes allowing the generation of multiple effector mutants with a single deletion event [45–47].

## Effectome functions depend on the plant target repertoire

To achieve the functional characterization of effectomes, we must take into account that effector functions are host dependent as they acquire their “functional sense” only in association with their plant cognate interactors (Fig 2B). Effectors tend to target multiple highly connected host proteins [22,26,27,48,49] but may also specifically interact with nucleic acids [50,51] or metabolites from the pathogen or the host [52–54]. Therefore, the function of a full effectome largely depends on the host target repertoire, or “targetome,” as well as on the interactions among its components (Fig 1C) as proposed [55]. Effector-mediated virulence is thus an emergent property resulting from interactions between a pathogen effectome and a targetome of susceptible host [56]. Effectome and targetome diversity should therefore be carefully considered since it should unravel the complexity and the diversity of the molecular mechanisms underlying pathogen virulence and plant susceptibility.

## Many lessons still to be learned from deconstructing effectomes

Effector polymutants are interesting resources to unveil functions of effector families [23,57–61] and effectomes [30,35,41]. Effector genes have to be deleted individually and sequentially. To date, the *Pseudomonas syringae* polymutant DC3000Δ36E is the only known mutant for a complex effectome in a plant pathogen [35]. In *P*. *syringae*, such effectome mutant has allowed significant discoveries not only in our understanding of the role of individual effectors but also most importantly in the identification of functionally redundant effectors [30] and the definition of minimal effectome functions required to become a plant pathogen [35,41,42]. To reach its full potential, we believe that future mutagenesis should aim at partial random deconstruction of effectomes using highly efficient tools such as CRISPR-Cas9 coupled to sensitive high-throughput pathogenicity assays on automated phenotyping infrastructures [62–65].

## Synthetic effectomes to understand how effectomes really function

Effectome mutants open the possibility not only to identify avirulence genes which recognition can be masked by other effectors [35–40] but also to reconstruct synthetic effectomes and test for their function. The choice of the receiver strain and the composition and size of the minimal effectomes to be tested in infection tests have to be carefully considered (Fig 2E). While natural effectorless strains often require the introduction of a functional effector secretion-translocation machinery [66,67], effectome mutants might still express and translocate yet unidentified effectors that could interfere with the characterization of synthetic effectomes [35,41]. Because of functional redundancy between effectors, functional synthetic effectomes can include only a portion of an original effectome (e.g., [41]). Effectors originating from other strains, species, genera, or even kingdoms could also be studied by such approaches as long as effector secretion-translocation happens (e.g., [68–70]). Though sometimes random [41], effector combinations have, up to now, been mostly based on gene families [60], gene clusters [41], or functional categories [30]. Yet, the combination of synthetic biology, next-generation sequencing technologies, and high-throughput phenotyping methods now opens the avenue for the generation of large random effector libraries to be tested in minimal strains and their functional characterization on host or nonhost plants (Fig 2F).

Similar limitations hindering effectomes characterization also apply to animal pathogens. Though these effectomes are also major virulence determinants (e.g., [71–74]) and the first effectome polymutant was generated in *Yersinia enterocolitica* [75], effectomes studies are also extremely limited in animal pathogens. We believe that the proposed holistic genetic approaches applied to effectomes should greatly advance our understanding of 2 basic questions: How do pathogens evolve and adapt to new hosts?
